# Anisotropic conduction in the myocardium due to fibrosis: the effect of texture on wave propagation

**DOI:** 10.1038/s41598-020-57449-1

**Published:** 2020-01-21

**Authors:** T. Nezlobinsky, O. Solovyova, A. V. Panfilov

**Affiliations:** 1 Ghent University, Department of Physics and Astronomy, Krijgslaan 281, 9000 Gent, Belgium; 2 Ural Federal University, Ekaterinburg, Russia; 3 Institute of Mathematics and Mechanics, Ural Branch of Russian Academy of Sciences, Ekaterinburg, Russia; 4 Institute of Immunology and Physiology, Ural Branch of Russian Academy of Sciences, Ekaterinburg, Russia

**Keywords:** Computational models, Arrhythmias

## Abstract

Cardiac fibrosis occurs in many forms of heart disease. It is well established that the spatial pattern of fibrosis, its texture, substantially affects the onset of arrhythmia. However, in most modelling studies fibrosis is represented by multiple randomly distributed short obstacles that mimic only one possible texture, diffuse fibrosis. An important characteristic feature of other fibrosis textures, such as interstitial and patchy textures, is that fibrotic inclusions have substantial length, which is suggested to have a pronounced effect on wave propagation. In this paper, we study the effect of the elongation of inexcitable inclusions (obstacles) on wave propagation in a 2D model of cardiac tissue described by the TP06 model for human ventricular cells. We study in detail how the elongation of obstacles affects various characteristics of the waves. We quantify the anisotropy induced by the textures, its dependency on the obstacle length and the effects of the texture on the shape of the propagating wave. Because such anisotropy is a result of zig-zag propagation we show, for the first time, quantification of the effects of geometry and source-sink relationship, on the zig-zag nature of the pathway of electrical conduction. We also study the effect of fibrosis in the case of pre-existing anisotropy and introduce a procedure for scaling of the fibrosis texture. We show that fibrosis can decrease or increase the preexisting anisotropy depending on its scaled texture.

## Introduction

The mechanical contraction of the heart is initiated by the propagation of electrical waves of excitation. Abnormal propagation of such waves can result in cardiac arrhythmias. Sudden cardiac death due to arrhythmia is one of the largest causes of death in the industrialized world, and therefore studies of the factors underlying wave propagation in the heart are of great importance. Electrical waves are generated by billions of interconnected cardiac cardiomyocytes. There are also other types of cells in the heart, with the majority being fibroblasts. The main role of fibroblasts is to maintain the structural and electro-mechanical integrity of the heart^1^ and to repair the heart after disease^2^. The fibroblasts are non-excitable cells, and therefore serve as obstacles to electrical wave propagation. In normal conditions, their effect on wave propagation is minimal. However, in many forms of heart disease and during aging the number of fibroblasts can substantially increase, which has pronounced effects on wave propagation. The increase of number of fibroblasts, fibrosis, is currently considered one of the most important arrhythmogenic conditions^3^. Experimental studies of mice hearts have shown that the inducibility of ventricular arrhythmias increases in a nearly linear fashion with the degree of fibrosis^4^. However, it has also been shown that not only the degree but also the texture of fibrosis substantially affects the propagation of electrical waves. There are several types of fibrosis, including interstitial, compact, patchy and diffuse^3^. In diffuse fibrosis, short non-conducting obstacles are mixed with cardiomyocytes. In interstitial and patchy fibrosis, there are long non-conducing obstacles mainly oriented along the myocardial fibers. Of these textures, the least arrhythmogenic is considered to be compact fibrosis, while the most arrhythmogenic are patchy and interstitial fibrosis, as these can cause large disturbances in wave propagation because of the zig-zag conduction between the various bundles^5^. Because the main feature of patchy and interstitial fibrosis is the presence of elongated obstacles, it is important to understand the possible effects of such elongation on wave propagation in cardiac tissue.

The systematic generation of fibrosis patterns with given textures in experiments is difficult and therefore other alternative methodologies are widely used to study the effect of fibrosis texture. One such method is mathematical modelling. Most of modelling papers are devoted to diffuse fibrosis and study its effect on wave propagation and the onset of arrhythmia^6–11^. The effects of fibrosis texture on wave propagation have also been studied in a few papers. In^12^ the effect of patch of fibrosis on extracellular electrograms was studied, and the main effect of fibroblasts was found to be via myocyte-fibroblast coupling. It was shown that such fibrotic tissue can form complex fractionated electrograms (CFAE). In^13^ the onset of CFAEs was related to the percolation threshold, and various fibrotic patterns with different densities were studied.

The most comprehensive study of the effect of tissue texture on wave propagation was performed by Pertsov^14^. The main focus of that study was on reproducing the effects of discontinuous conduction on the maximal depolarization rate (*dV*∕*dt*)_*max*_. In a low - dimensional model of cardiac tissue it was shown that the texture of inexcitable elongated obstacles can produce a directional difference in (*dV*∕*dt*)_*max*_, which should be absent in the case of continuous propagation. This effect becomes pronounced if the parameter *L*∕*W* has a value of 2–3, where *L* is the length of the obstacle and *W* is the spatial length of the wavefront. In addition, it was noted that a spatially periodic texture formed of elongated obstacles can create anisotropic propagation by slowing the condition in the transversal direction. It was noted that anisotropy can be controlled by an increase in the length of the obstacle *L*. This is a very interesting observation relating texture and anisotropy of the tissue. However, this effect was only studied using low - dimensional models of cardiac tissue, where the ratio of the front length and the duration of the pulse is normally much lower than in real tissue. In addition, the paper^14^ provides only one example of the effect for a given periodic structure. More recently a study by Jacquemet and Henriquez^15^ showed in a canine atrial model that microscale obstacles representing collagenous septa cause significant changes to the electrogram waveforms and transversal conduction velocity.

Overall, we can emphasize that most modelling research is devoted to the effects of diffuse fibrosis only and that the effects of fibrosis texture are largely under-studied. In addition, as most important changes in texture originate with the development of elongated obstacles, the first step is to study the effects of such obstacle elongation. The aim of this paper is to study in detail effects of randomly generated textures with various lengths and percentages of obstacles on wave propagation in cardiac tissue.

## Methods

### Mathematical model

To describe the electrophysiological properties of the isotropic cardiac tissue, we used a 2D mono-domain formulation^16^:

1 $$\begin{array}{ccc}Cm\frac{\partial u}{\partial t}=D{\nabla }^{2}u-{I}_{ion}, &  & \end{array}$$

where *u* – is the transmembrane voltage, *D* – is the diffusion matrix for anisotropic tissue and constant for isotropic tissue, *I*_*ion*_ – is the sum of all transmembrane ionic currents. The ionic current can be represented using a low dimensional phenomenological models as in papers^14,16^, or using biophysically detailed representation of the ionic currents. In this paper we use detailed representation of *I*_*ion*_ taken from^17,18^.

Initial conditions were set at the rest potential for the cardiac tissue. Boundary conditions were formulated as the no flux through the boundaries:

2 $$\overrightarrow{n}\nabla u=0,$$

where $\overrightarrow{n}$ - is the normal to the boundary.

We performed partial differential equation (PDE) calculations following^19^ and used the finite-difference scheme with the five-point stencil. The parameters of discretisation were 0.25 mm for the spatial step and 0.02 ms for the time step. The fibrosis part of the medium was simulated as non-conducting inexcitable obstacles was and processed in the same way as the boundaries (no flux).

### Fibrosis pattern

To create the desired fibrosis patterns, we considered a 2D finite difference mesh as a matrix with the combination of two types of elements: excitable myocardial elements and inexcitable fibrosis elements. The fibrosis elements were uniformly distributed through the mesh, and the rest of the mesh was represented by the myocardial elements. In this study, the only fibrosis texture of unidirectional elongated obstacles ("striae” pattern) was simulated as a set of parallel linear segments of the same length. The obstacle length was then varied to study the effects of increased elongation of the striae. This speculative fibrosis pattern implies a sequential row-by-row revision of all matrix elements. We tried two approaches. In the first approach, we start form the 1st row and the 1st column (element (1, 1)) and assign a fibrotic element of the length *n* in the horizontal direction with a probability *p*. If the fibrotic element is assigned, we go to the element (n + 1, 1) and repeat the procedure. If it is not assigned, we go to the element (2, 1) and repeat the procedure. After that we go to row 2, 3, etc. Although this algorithm works good, the actual percentage of fibrosis generated in such way is below *p* and for the large fibrosis percentages the deviation can be quite substantial. Thus here, we always need to assess the real percentage of fibrosis for each texture. We have also tried another approach. We subdivide each row into blocks on length *n* and assign it as fibrotic with a probability *p* (using the Mersenne twister algorithm). In such approach, we always get a real fibrosis percentage very close to *p*. We performed test runs and found that both approaches give the same qualitative and very close quantitative results (within an error due to variation of the texture). However, in the case of the first approach generation of textures with exactly the same percentage of fibrosis is more difficult. As we are interested to compare different textures with the same fibrosis percentage, we decided to use approach two. Note, however, that we also performed a set of simulations using approach one which produced the same qualitative result.

### Shortest propagation path

The shortest paths of excitation propagation between the opposite sides of the fibrosis matrices are used in the results section to demonstrate the zig-zag propagation for the fibrosis texture with elongated obstacles. To show the possibility of simply bypassing obstacles, we used a primitive cellular automata simulation where a non-activated cell is activated if at least one of its four side neighbours is activated in the previous step of calculations. We then built a simple undirected graph connecting neighbouring active cells for each fibrosis matrix. Using this graph, the shortest propagation path from the initial source point on the stimulated border of the tissue to the first activated point on the opposite border of the tissue was found and its length calculated.

### Wavefront detection

**Figure 1:**
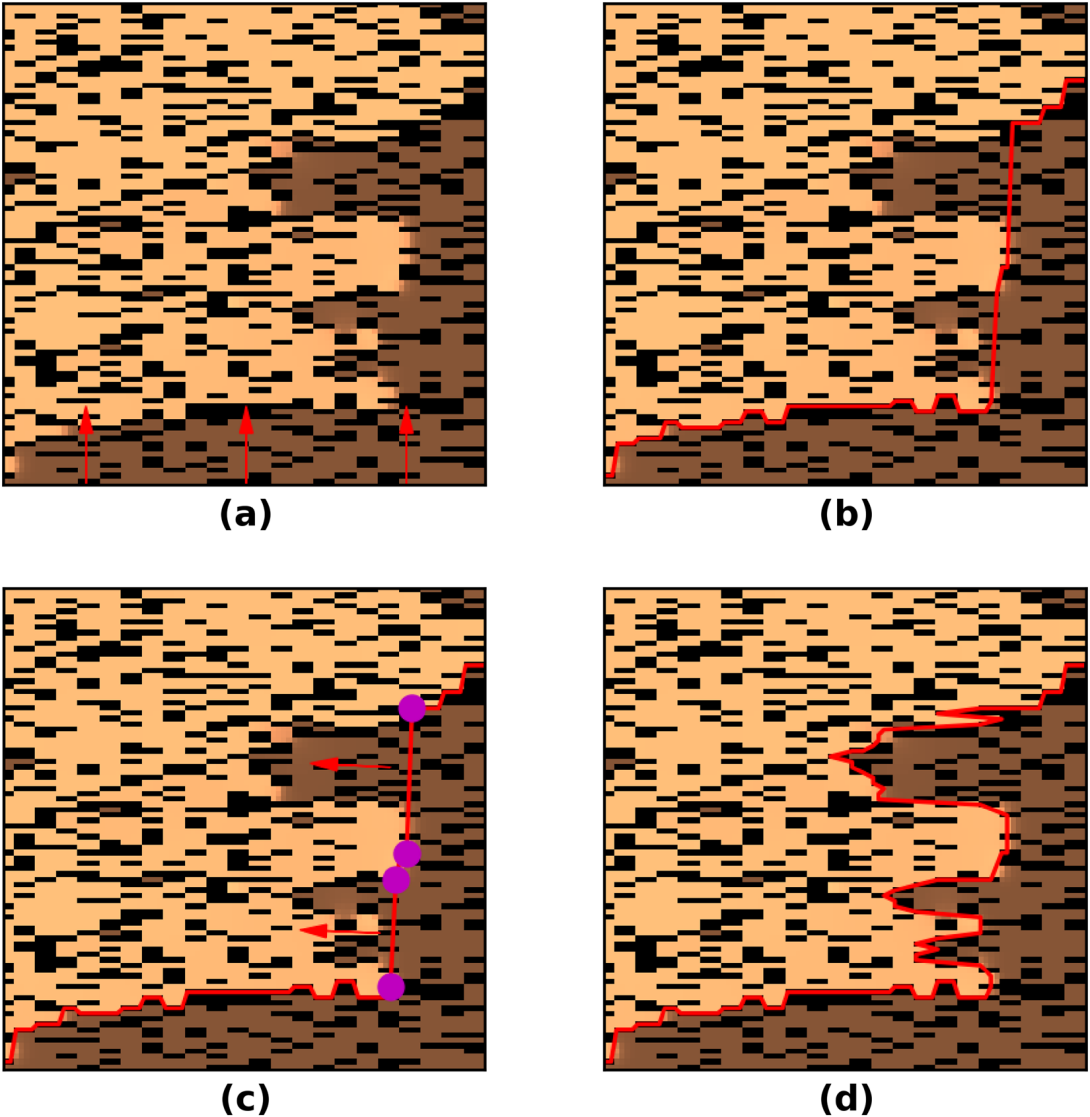
A part of the texture with elongated fibrosis, wave propagation comes from the upper side of the square. The light color indicates activated cells, the dark color (brown) indicates non-activated cells and the black color indicates fibrosis. The wavefront is marked as a red line and the gap locations are marked at the edges with the purple color.

In the results section we introduced wavefront length as a characteristic of wavefront complexity due to the passage through the texture of fibrosis. We used a "divide and conquer" algorithmic approach to determine the wavefront in different variations (Fig. 1). In the fist step (Fig. 1a), from each node on the down side of the lattice we move to the first activated node where *u* ≥ −10 *mV* (boundary nodes on the activated area) in the direction opposite to the wave propagation. In the second step, we connect the boundary nodes in a line to complete the first iteration (Fig. 1b). In the third step (Fig. 1c), we search for gaps in the border line longer than 2 mm. In the last step (Fig. 1d), we repeat the search for activated nodes in the direction perpendicular to the gap lines and fill the rest of the boundary. This procedure can be repeated recursively while at least one gap remains in the wavefront.

### Anisotropy estimation

**Figure 2:**
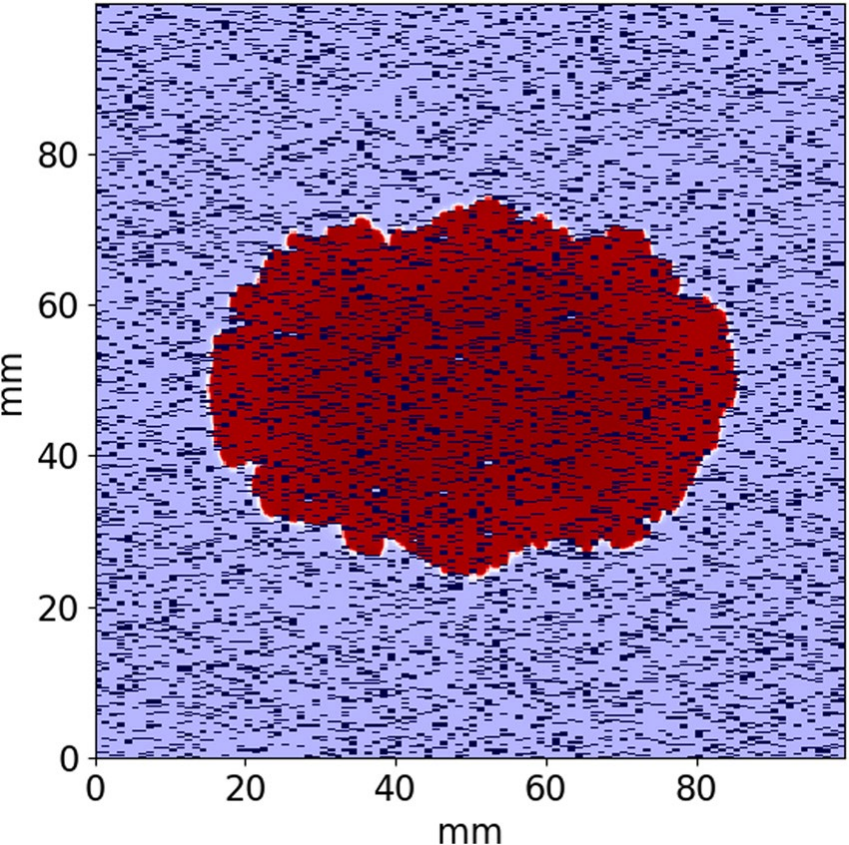
An example of point stimulus and elliptic wave propagation (red) for 15% fibrosis area with the obstacles size 0.25 × 0.75 mm.

In the results section we described an approach to evaluate the propagation anisotropy for a point-like source of stimulation (Fig. 2). The velocity vector in each point of the elliptic wavefront can be obtained from:

3 $$\overrightarrow{\upsilon }=\left(\begin{array}{ll}{D}_{1} & 0\\ 0 & {D}_{2}\end{array}\right)\left(\begin{array}{l}{\upsilon }_{iso}{\rm{\cos }}\alpha \\ {\upsilon }_{iso}{\rm{\sin }}\alpha \end{array}\right)$$

where *υ*_*iso*_ - is the velocity of the planar wave in the isotropic medium, *D*_1_, *D*_2_ - are the scaling factors for wave velocity along and across the obstacle for planar waves and *α* - is the angle of the wavefront point in the polar coordinate system located at the stimulation point.

We then used a non-linear least squares to fit the equation 3 to the simulated data with respect to parameters *D*_1_ and *D*_2_ to estimate the anisotropic property of the texture.

## Results

**Figure 3:**
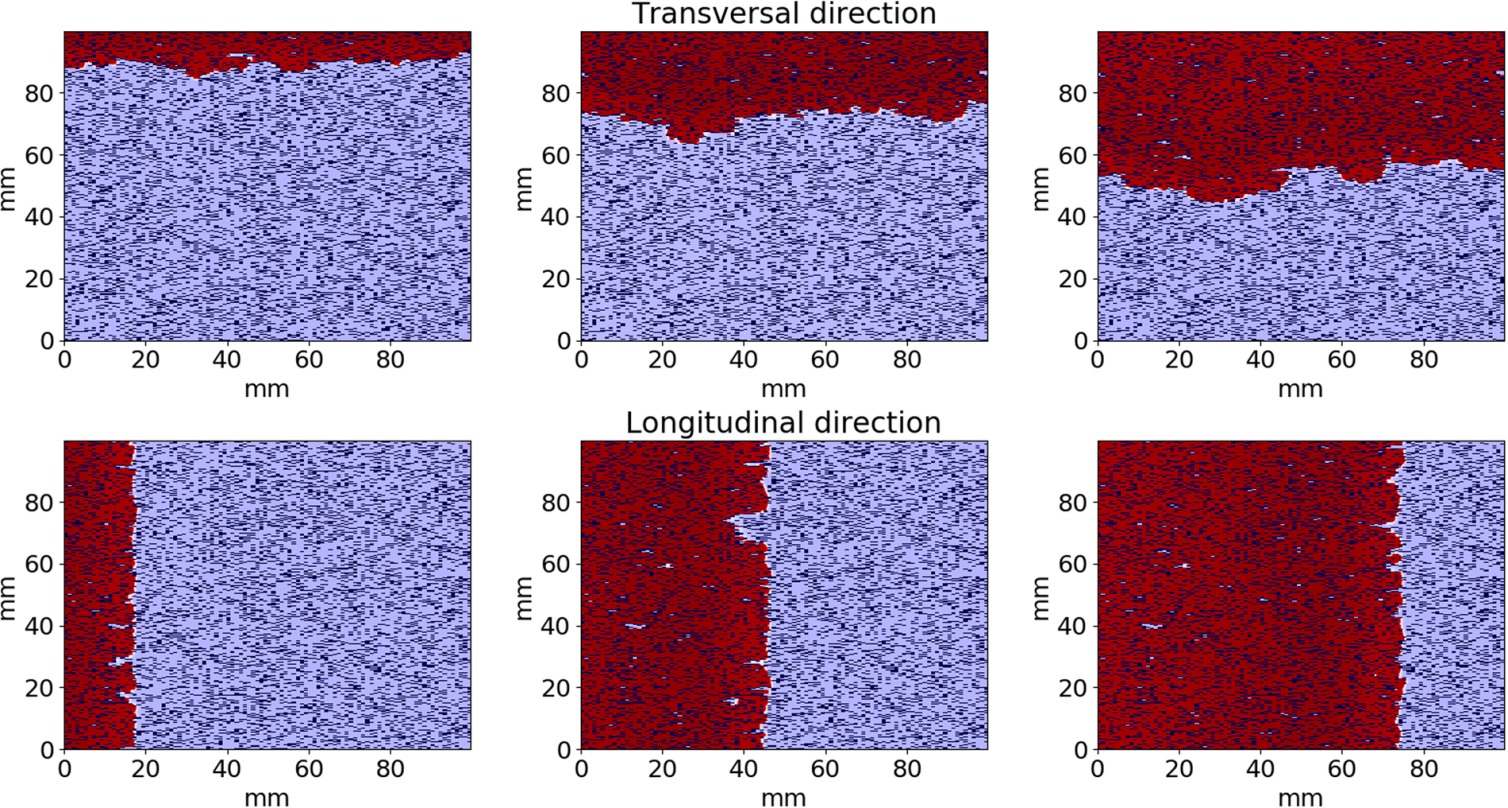
An example of wave propagation through the fibrosis texture across (upper) and along (lower) the obstacles orientation for t = 25, 70, 115 msec. Fibrosis area is 20% and the fibrosis (obstacles) size is 0.25 × 1 mm. Activated areas are shown in the red color.

We studied the propagation of the excitation wave for fibrosis textures with various obstacle lengths. We considered various patterns of obstacles with size ranging from one node (diffuse fibrosis) to four nodes. All elongated obstacles were oriented horizontally. We varied the fibrosis percentage from 0% to 35% and studied the wave propagation along the orientation of the obstacles (longitudinal) and across them (transversal). A representative simulation of the wave propagation is shown in Fig. 3. We see that the wavefront has a complex shape due to interaction with obstacles. We also see that the wave propagates along the obstacles faster than across them. For example, 115 ms after stimulation the wave propagates at a distance of approximately 80 mm for the longitudinal propagation. However, for the transversal propagation the wavefront is located at about 60 mm from the stimulated side of the tissue.

### Wavefront velocity depends on fibrosis fraction and direction

**Figure 4:**
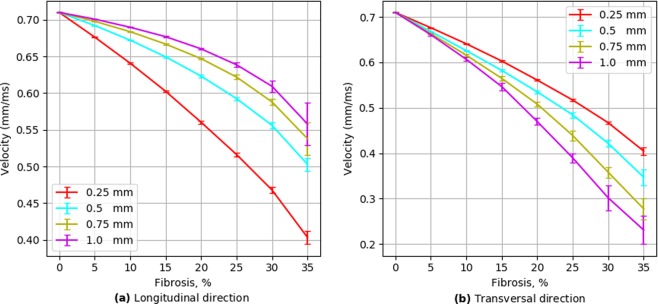
Wavefront velocity depending on fibrosis area in two wavefront directions. Error bars show the standard deviation of the wavefront velocity in 20 fibrosis pattern samples for each obstacle length.

 Figure 4 shows the dependency of the conduction velocity on the percentage of the fibrosis for textures from one to four nodes long. As expected, the velocity significantly decreases with an increase in the fibrosis percentage. Obviously, for the diffuse fibrosis the velocity in the longitudinal and transversal directions is the same (the red line). We use it as a reference to compare with that velocity in the textures with elongated obstacles. We see that the elongation of obstacles unexpectedly has the opposite effect on the longitudinal and transversal conduction velocity. Indeed, for the longitudinal propagation the conduction velocity increases with an increase in obstacle length, while for the transversal propagation the velocity decreases. Therefore, the texture of fibrosis substantially affects the conduction velocity and makes propagation anisotropic, similar to^14^.

**Figure 5:**
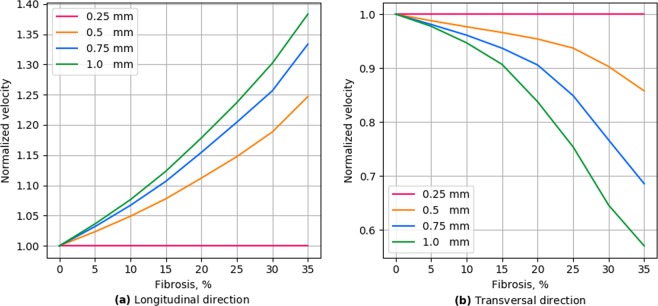
Wavefront velocity normalized by the values of diffuse fibrosis.

**Figure 6:**
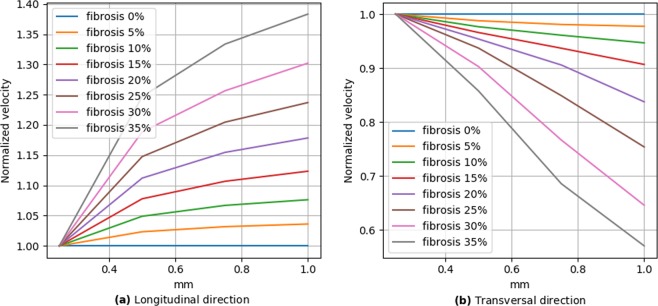
Wavefront velocity normalized to values of diffuse (size = 0.25 mm) fibrosis depending on fibrosis length in two wavefront directions.

To further quantify the effects of obstacle elongation, we plotted the ratio of the velocity for elongated obstacles to the velocity for the diffuse fibrosis (Fig. 5). We compared velocities for the same amount of fibrosis but for diffuse and elongated cases and can regard it as a pure effect of elongation. We see that the effect of elongation almost linearly increases with the percentage of fibrosis. Figure 6 shows that the effect of elongation almost linearly depends on the obstacle size for the transversal direction of propagation, while it has a more complex dependency with a tendency to saturation for the longitudinal direction of propagation.

Thus, our results clearly show that the texture of the fibrosis has a substantial effect on the propagation velocity and makes propagation anisotropic by decreasing the transversal and increasing the longitudinal velocity with obstacle elongation. The effects increase with the percentage of the fibrosis and with the elongation of the obstacles.

**Figure 7:**
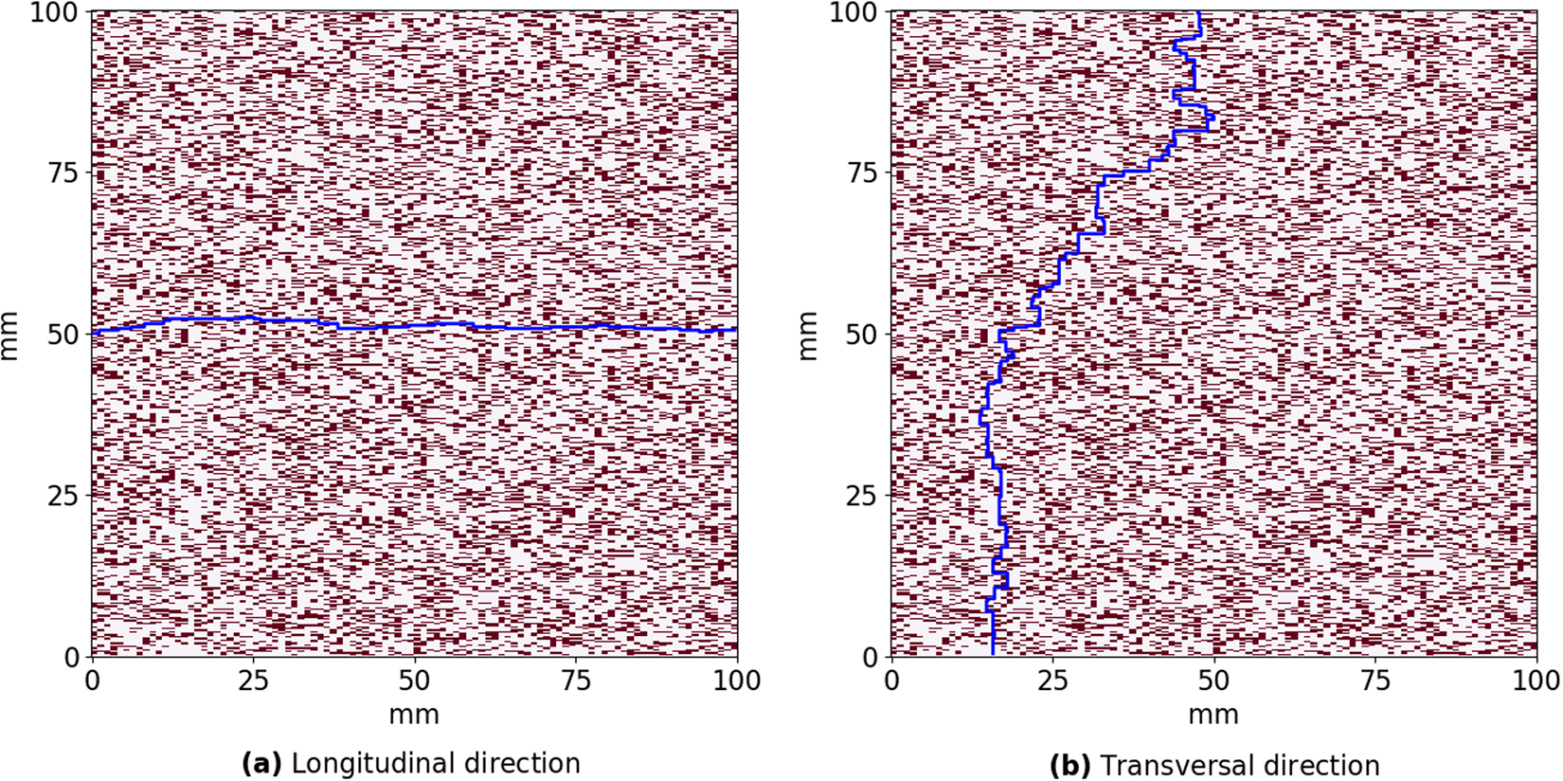
The shortest path from point (0, 50) to the opposite side in longitudinal direction (**a**) and from point (47, 0) to the opposite side in transversal direction (**b**) for the same matrix with 30% fibrosis area and 0.25 × 1 mm fibrosis size.

**Figure 8:**
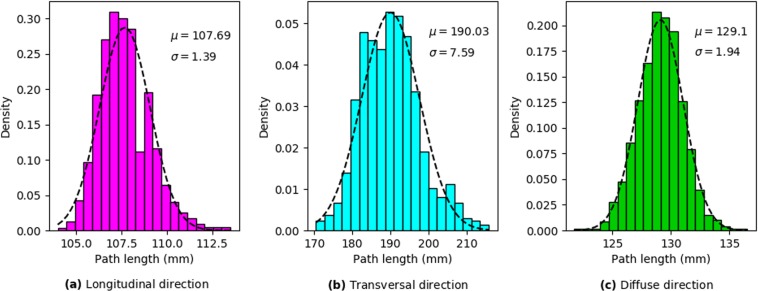
Normalized histograms of the shortest path for the longitudinal propagation (**a**) and transversal propagation (**b**) for 0.25  ×  1 mm length fibrosis and for diffuse fibrosis (**c**). 5000 trajectories calculated in 20 fibrosis matrices of 30% are used for each histogram.

From a general point of view, this result is not trivial. Indeed, if we consider tissue with a given percentage of fibrosis, for example, 30%, it means that for all textures, 30% of all cells are non-conducting. However, as elongated obstacles contain several non-conducting cells, the total number of obstacles in tissue decreases with the obstacle length. For example, the total number of obstacles with four nodes will be four times less than for diffuse fibrosis with the same percentage. Why does such a smaller number of obstacles substantially decreases the wave propagation for the transversal propagation and substantially increases it for the longitudinal one. We can find a general answer to this question in papers^5,14^. In general, the mechanism of slowing the propagation occurs because the wave needs to go around obstacles, which increases its traveling path and thus decreases the velocity. If the obstacles are large, the traveling path will substantially differ from a straight line and result in zig-zag propagation^5^. To quantify this effect, we performed the following additional simulations. We started with a given point at the boundary of the tissue and found the shortest geometrical path to the opposite boundary along the myocytes network. In Fig. 7a, we show the line of the minimal length that goes from a given point at the left boundary to the right boundary along the white cells (conducting) in a given texture. Figure 7b shows a similar path from a point at the upper boundary. We see that for these two situations that would occur for the longitudinal and transversal waves the paths substantially differ, and propagation across the obstacles results in a more complex and longer trajectory. We quantified this effect for a fibrosis texture of 30% with an obstacle length of four nodes and for diffuse fibrosis of one node length. In particular, in every texture matrix we found the shortest path for each starting point at the upper boundary to quantify the transversal propagation and for each starting point at the left boundary to quantify the longitudinal propagation. The distributions for the zig-zag path length are shown in Fig. 8. We see that for the obstacles of four nodes long the average path for the transversal propagation is 190 mm, for the longitudinal propagation it is 108 mm and for the diffuse fibrosis it is 129 mm. Interestingly, for that particular texture the ratio of conduction velocities in the longitudinal and transversal directions is 1.95, which is reasonably close to the ratio of average path lengths (190/108 = 1.76).

In Fig. 8, we evaluated the shortest path using geometrical connectivity arguments, as in^20,21^. However, in addition to connectivity, the path of the wave is determined by the varying electrotonic load (source-sink relationship), which can cause a local block for propagation even in the case of geometrical connectivity and thus change the path of the wave^22^. To quantify it we performed additional simulations. These simulations are similar to those performed in Fig. 8, however, instead of finding the shortest geometric path, we initiated a real wave by point stimulation at one boundary and determined its shortest propagation path to the opposite boundary. In doing so, we obtained the following results. For the propagation in the longitudinal direction the average path was 110 ± 2.3 mm and for the propagation in the transversal direction the average path was 219 ± 10.1 mm. These values are longer than those for the geometric connectivity path shown in Fig. 8, which were 107.7 ± 1.4 mm and 190 ± 7.6 mm correspondingly. Thus, we see that the source-sink relationship does influence zig-zag propagation patterns and its effect is different for the longitudinal and for the transversal propagation. For the longitudinal propagation the path elongation due to the source-sink relationship is 2.1%, while for the transversal propagation, the path elongation is 15.3%. Also note that the ratio of the average path lengths now becomes 219/110 = 1.99, which almost exactly coincides with the ratio 1.95 of the conduction velocities in the longitudinal and transversal directions for that particular texture. Thus, we can conclude that the presence of longer obstacles increases the geometric length of the trajectory due to zig-zag propagation and the source-sink relationship further enhances the effect due to the larger influence on the transversal propagation.

### Fibrosis texture changes pre-existing anisotropy

**Figure 9:**
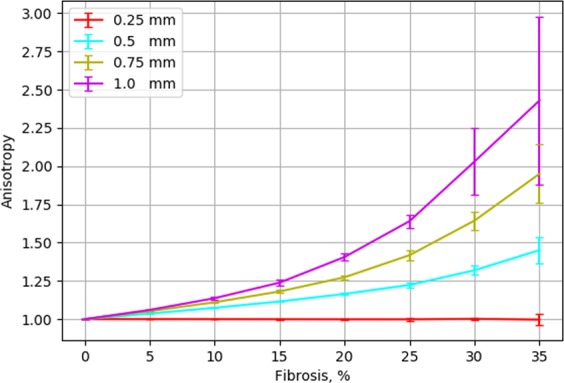
Ratio in conduction velocity between the longitudinal and transversal wavefront direction as a characteristic of the fibrotic texture anisotropy. Error bars show the standard deviation of the velocity’s ratio for 20 repeats with unique fibrosis matrices.

**Figure 10:**
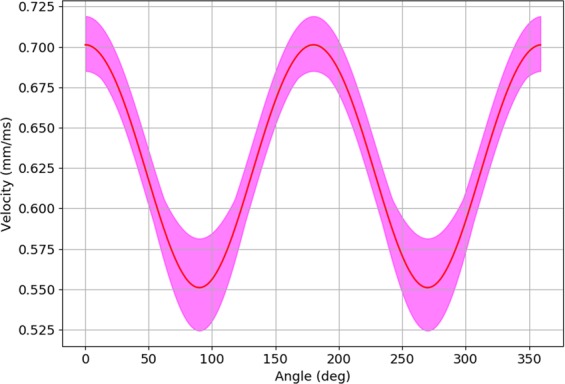
Wavefront velocities depending on the polar angle for 20 simulations with the same 15% fibrosis matrix with the obstacles size 0.25 × 1 mm. The red line shows the average value of the velocity, while the purple area limits its maximum and minimum value.

The difference in the velocity of wave propagation in the longitudinal and the transversal directions obviously leads to anisotropic wave propagation. Figure 9 shows anisotropy as the function of the fibrosis percentage and obstacle length. We see that both fibrosis density and the length of the obstacle increases the anisotropy of the tissue. We have also studied if the anisotropy obtained via such a mechanism has an anglular dependency as a conventional elliptic anisotropy. Figure 10 shows the dependency of the wave velocity on the direction for the point stimulus and its elliptic fit. On average, we can consider the anisotropy as being of the elliptic type; however, we see large local deviations due to the heterogeneity of the local fibrosis texture.

**Figure 11:**
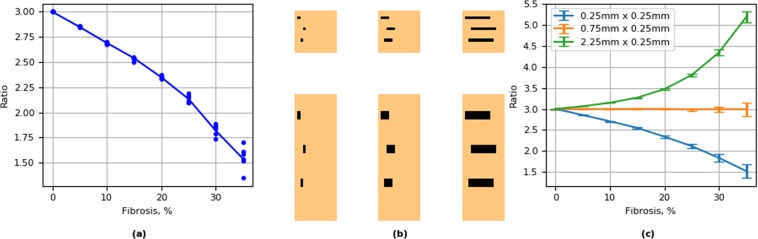
Effect of fibrosis texture on pre-existing anisotropy of cardiac tissue. (**a**) effect of diffuse fibrosis. Points show predictions for anisotropy based on results of Fig. 9 (further explanation are in the text); (**b**) schematic representation of normal (the upper pannel) and scaled (the lower pannel) textures; (**c**) Effect of fibrosis on anistropy for textures generated by obstacles of various size.

We studied the effects of the propagation of the excitation wave for fibrosis textures with various obstacle lengths in anisotropic cardiac tissue. For that we performed simulations in tissue with parallel horizontal fibers with anisotropy 3:1, with respect to velocity (the ratio of the diffusion coefficients Dx/Dy = 9:1). We first studied the effect of diffuse fibrosis induced by the inexcitable blocks of 0.25 mm  ×  0.25 mm on the anisotropy (Fig. 11a). We see that the diffuse fibrosis, which previously had an equal effect on the longitudinal and transversal velocity and thus did not induce any anisotropy (Fig. 9), unexpectedly resulted in reduction of the existing anisotropy. We see that for the diffuse fibrosis of 35%, anisotropy reduced almost two times (from 3:1 to 1.54:1). In order to understand it, let us consider Eq. (1). In our case for parallel horizontal fibers with anisotropy 3:1 the Laplace operator can be rewritten as:

4 $${\nabla }^{2}u={D}_{x}{\partial }^{2}u/\partial {x}^{2}+{D}_{y}{\partial }^{2}u/\partial {y}^{2},$$

where *D*_*x*_ and *D*_*y*_ are the diffusion (coupling) coefficients along and across the fibers and *D*_*x*_∕*D*_*y*_ = 9. It is easy to see that expression (4) can be rewritten as:

5 $${\nabla }^{2}u={D}_{x}({\partial }^{2}u/\partial {x}^{2}+{\partial }^{2}u/\partial {Y}^{2})$$

where

6 $$Y=y* \sqrt{{D}_{x}/{D}_{y}}.$$

Expression (5) can viewed as follows. It describes the Laplacian in an isotropic tissue with the diffusion coefficient *D*_*x*_ in the new coordinate system (*x*, *Y*) (6). This new coordinate system is just a rescaling of the original coordinate system. In our case, it is just stretching of the y-axis by $\sqrt{{D}_{x}/{D}_{y}}=3$ times. This means that one can choose to interpret the effects of anisotropy geometrically as rescaling. Let us apply this approach to explain the results shown in Fig. 11a. Here we have pre-existing anisotropy with respect to wave propagation velocity *v*_*x*_/*v*_*y*_ = 3. On top of it, we have the diffuse fibrosis generated by the squared obstacles of the size of 0.25 × 0.25 mm. Let us consider this texture in the new coordinate system (*x*, *Y*), in which wave propagation is isotropic. In this system, the squared obstacle 0.25 × 0.25 mm will become an obstacle of the size 0.25 × 0.75 mm, and our problem of wave propagation in anisotropic tissue with diffuse fibrosis will be equivalent to the problem of wave propagation in isotropic tissue with small vertically oriented rectangular obstacles. We already performed such simulations for obstacles of the size 0.75 × 0.25 mm (see Fig. 9 the light green line) and found that they induce anisotropy. However, in simulations of Fig. 9, such obstacles were oriented horizontally. Because the obstacles are now oriented to the *Y* direction, the results shown in Fig. 9 give us the ratio *v*_*Y*_/*v*_*x*_ depending on the percentage of fibrosis. Now let us find how such textures will affect anisotropy in our original system (*x*, *y*). As ${v}_{Y}={v}_{y}\sqrt{{D}_{x}/{D}_{y}}$ the anisotropy in our original system ${v}_{x}/{v}_{y}={v}_{x}/{v}_{Y}\sqrt{{D}_{x}/{D}_{y}}=3{v}_{x}/{v}_{Y}$ where *v*_*x*_∕*v*_*Y*_ is the inverse value of the anisotropy given by the light green line in Fig. 9. We took that anisotropy values for 48 representative examples of simulations performed in Fig. 9 and placed them as points to Fig. 11a. We see a perfect match of the predicted and simulated data. Thus, we can conclude that in order to find the effect of the texture of fibrosis on anisotropy, it maybe helpful to rescale this texture based on the tissue’s pre-existing anisotropy. In our case it means the following. Obstacles elongated along the fibers with the size 0.75 × 0.25 mm will be equivalent to the squared obstacles with the size 0.75 × 0.75 mm (Fig. 11b); therefore such texture should not affect the anisotropy. Only if the ratio of the length to the width of the obstacles will be larger than $\sqrt{{D}_{x}/{D}_{y}}=3$ (e.g. 2.25 × 0.25 mm) it will increase the pre-existing anisotropy. The results shown in Fig. 11c confirm this simple analysis.

### Wavefront length

**Figure 12:**
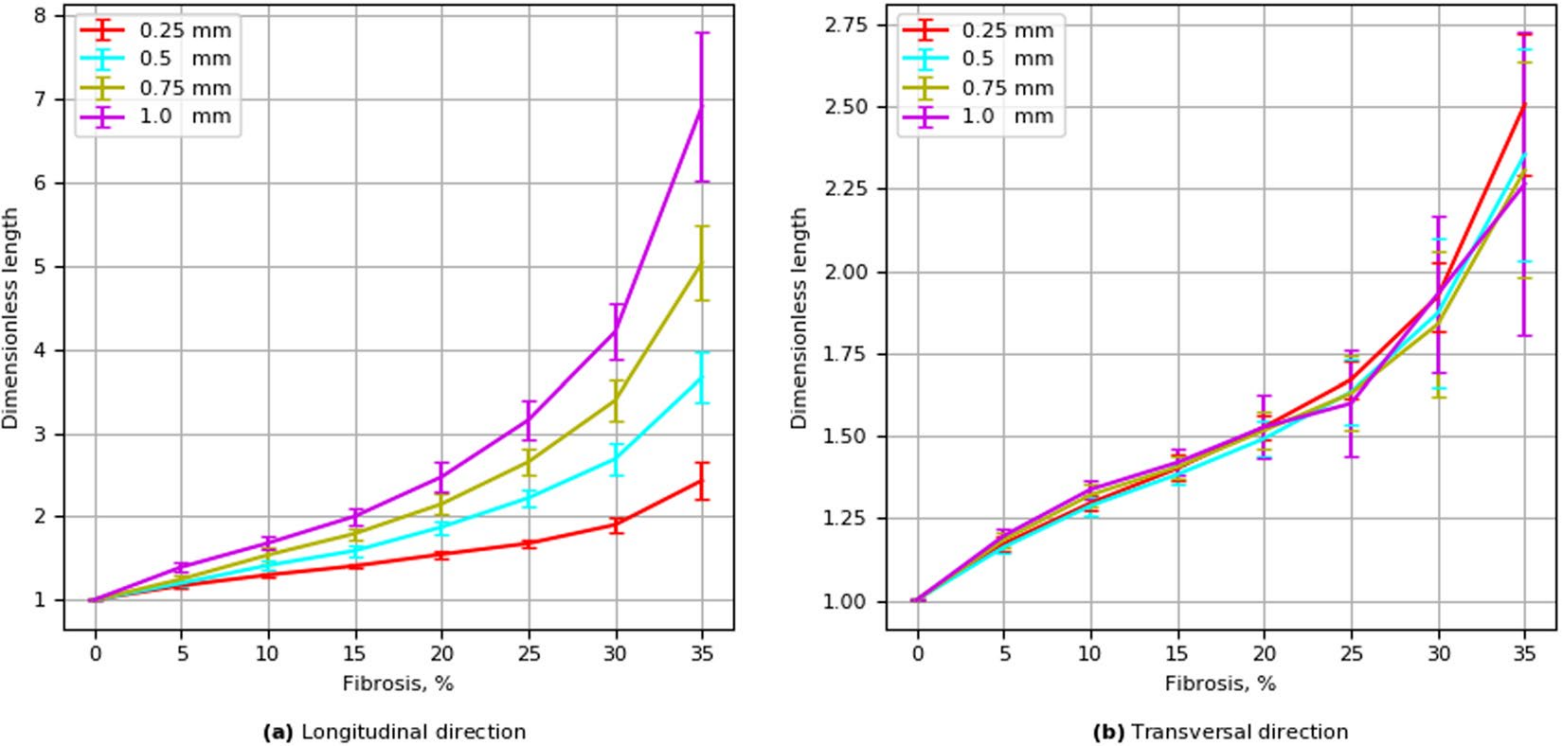
Dependence of the wavefront t-length on fibrosis density for longitudinal and transversal wave propagation. Error bars show the standard deviation of the t-length for 20 simulations for each fibrosis texture.

During wave propagation in the fibrotic tissue, the shape of the wavefront is complex and differs substantially from the plane wavefront. Such a complex wavefront shape is another qualitative characteristic that has an effect on fibrosis. To quantify such elongation, we used the dimensionless index t-length, which is the length of an isopotential line at a voltage level of  − 10 mv during the depolarization phase divided by the spatial size (width) of the tissue (Fig. 12). We used the term t-length to distinguish it from the front length introduced in^14^, which was measured orthogonal to the level line. We see that for the transversal propagation the t-length increases with an increase in the fibrosis, but, it almost does not depend on the obstacle length. This is an unexpected result, as the velocity of the wave in that parameter range shows a strong dependency on the obstacle length. For the longitudinal propagation, the t-length increases both with an increase in the fibrosis area and in the length of the obstacle. In addition, the t-length for the transversal propagation for higher persentages of fibrosis is from two to four times shorter than for the longitudinal propagation.

## Discussion

In this paper, we performed a detailed study of the effects of fibrosis texture on wave propagation in cardiac tissue. In particular, we are interested in the effects of obstacle elongation. Of course, this is a simplification of the real situation. It can be viewed as an attempt to describe the transition from diffuse fibrosis to interstitial fibrosis^5^. As most previous modelling research dealt with diffuse fibrosis only^6–11^ consideration of obstacle elongation makes, in our view, the next logical step toward more realistic fibrosis textures.

We show that the elongation of obstacles has a substantial effect on the waves. It results in anisotropic wave propagation in which the velocity of the longitudinal propagation increases with increase in elongation, while the velocity of the transversal direction decreases. The extent of the effect increases with the fibrosis percentage and with the obstacle length. The idea that the texture of the tissue can induce anisotropy was proposed by Pertsov^14^. He explained it by a decrease in the transversal velocity and illustrated the effect on few a periodic structures in a two-variable FitzHugh–Nagumo model of cardiac tissue. Here, we performed extensive simulations for various randomly generated fibrosis textures of various density utilizing a modern ionic model of human cardiac tissue and further quantified the effects. We showed that the anisotropy becomes essential only for a high degree of fibrosis (20% or more) and that both longitudinal and transversal velocities of propagation in striated fibrosis are affected compared to diffuse fibrosis. We found that the effects of the obstacle length are opposite; the longitudinal velocity increases and the transversal velocity decreases with increases in the obstacle length. We also quantified the mechanism responsible for the anisotropy in the fibrotic tissue and provided a first quantitative estimate of the effect of tissue texture on the zig-zag propagation and quantified effects of geometric connectivity and source-sink relationship.

In^14^, Pertsov also noted that the effect of obstacles can be essential if the obstacle length *L* is comparable with the length of the wavefront *W*. In our case, the front length is about 1 mm (or four node length) and in Fig. 8 we indeed see that in the case of four-node obstacle length we observe anisotropy above 2:1 for fibrosis of more than 30%. However, note, that even for the obstacles of 0.5 mm the effect can still be substantial if the fibrosis percentage is large. This means that the effect of the factor *L*∕*W* also depends on the percentage of fibrosis.

We have also studied the effect of texture of fibrosis on a pre-existing anisotropy of cardiac tissue. We show that fibrosis can either increase of decrease the anisotropy. Interestingly enough we show that diffuse fibrosis should decrease the anisotropy and that in order to evaluate the effect of fibrosis it may help to rescale its texture. Note, however, that practical application of such rescaling is not straightforward. Simple rescaling will work only for the case of parallel fibers. Also, as normally the anisotropy ratio in the absence of fibrosis is not known, such rescaling should be based on some reasonable assumptions. Evaluation of the effect of fibrosis in the case of complex anisotropy can potentially be performed using the methods of Riemannian geometry developed in^23–25^. One way to do that, could be to estimate the local change of anisotropy based on local rescaling of the texture (similar to what we did for Fig. 11a), and then translate it to the eigen values of the diffusion matrix. After that the effects can be evaluated using approaches from^23–25^.

The results of the effects of fibrosis texture in anisotropic tissue presented here are obtained for the fibrosis represented as a set of the finite size inexcitable obstacles. If, however, the interstitial fibrosis is modelled using approach^26^ as an internal boundary condition, the thickness of the obstacle will be zero. Therefore, although rescaling will still work here, it should show that such decoupling along the fibers will always increase the pre-existing anisotropy. Note, however, that as real fibrotic textures do have some thickness, even for interstitial fibrosis, it would be interesting in the future to study the effect of the texture based on detailed anatomical data.

We also studied the effect of fibrosis texture on the t-length of the wavefront. We unexpectedly found that in the transversal direction of propagation the t-length does not depend on the obstacle length and is affected by the percentage of fibrosis only. In contrast, in the longitudinal direction of propagation the t-length substantially increases with increases in obstacle length. The exact mechanism of this effect is still not clear. What we frequently saw, is that for longitudinal propagation there is a large local dispersion in the propagation velocity in the adjacent tissue regions that results in some local areas of delayed activation compared to neighbouring tissue. We think that the elongation of the obstacles creates a difference in wave velocity in the direction of obstacles (along the obstacles), while for the transversal propagation (across the obstacles) velocity is more spatially uniform, resulting in a more straight front of smaller length. However, we were not able to quantify this effect.

Our approach has several limitations. We studied fibrosis as multiple inexcitable obstacles. However, fibroblasts also produce biologically active molecules that can affect the electrical properties of myocytes^27^. It would be interesting to study the combined effects of fibrosis and ionic remodelling on wave propagation. We considered 2D isotropic tissue with simple rule-based fibrosis textures. In real situations, the fibrosis is three dimensional and has a more complex spatial structure. It would be interesting to perform studies on realistic textures of cardiac fibrosis in the human heart similar to those from^28^ obtained for 3D tissue^29^ using computational approaches presented in study^30,31^.

We studied effects of fibrosis without taking into account the detailed shape of fibroblasts and myocytes. It was shown^32^ that microstructural shape and connections of myocytes create additional pathways of current flow that enhance both the longitudinal and the transverse propagation. Also it was shown in^33^ that detailed representation of the cell shapes in virtual cell cultures can substantially affect the velocity of wave propagation and anisotropy and create the conduction pathways in the tissue^34^. It would be interesting to study how the shape and configuration of myocytes can affect the results obtained in this paper. Studies by Jacquemet and Henriquez^15^ indicate that also in detailed models representing the call shape increasing the length of collagenous septa leads to similar effects on decreasing velocity for transversal propagation as in our paper. Thus we expect that qualitatively the results obtained in our paper will be similar when a detailed shape of myocytes and fibroblasts will be taken into the account. However, we can still observe essential quantitative changes for the given degree of fibrosis.

We did not study the effect of pacing frequency on wave velocity (CV restitution). We expect that a higher frequency will act in a similar way to increased fibrosis percentage and thus just shifts most of the dependencies. However, these studies can easily be performed using our methodology and will be included in a subsequent study.

In this paper, we considered fibroblasts as inexcitable cells that are electrically uncoupled from myocytes. It was shown that in some cases fibroblast-myocyte coupling can occur and that this can affect the velocity of wave propagation^35^. It will be interesting to study if the texture of fibroblast-myocyte coupling has additional effects on wave propagation velocity and anisotropy of the tissue.

We have studied the effects in a TP06 model of human cardiac tissue. It would be interesting to see if the same effects are also present in other models of human cardiac cells, such as^36,37^.

In conclusion, in a model for human ventricular cells we show that elongation of inexcitable inclusions characteristic to some types of fibrosis induces anisotropy of cardiac tissue. This effect depends on the fibrosis percentage. For example, in the studied conditions, anisotropy 1.5 can be induced in initially isotropic tissue by the obstacles of the length of 1 mm if the fibrosis percentage is more than 22%. Anisotropy occurs due to zig-zag propagation, which is mainly determined by the texture’s geometric factors. The source-sink relationship has a secondary effect on it. In the case of pre-existing anisotropy it maybe helpful to rescale the texture to see the effects of fibrosis more clear. For example, diffuse fibrosis is predicted to decrease pre-existing anisotropy.
